# DOTA Conjugate of Bisphosphonate and PSMA-Inhibitor: A Promising Combination for Therapy of Prostate Cancer Related Bone Metastases

**DOI:** 10.3389/fnume.2022.892147

**Published:** 2022-06-29

**Authors:** Tilmann Grus, Hanane Lahnif, Nicole Bausbacher, Matthias Miederer, Frank Rösch

**Affiliations:** ^1^Department of Chemistry-TRIGA Site, Johannes Gutenberg University, Mainz, Germany; ^2^Department of Nuclear Medicine, University Medical Center Mainz, Mainz, Germany

**Keywords:** PSMA, bisphosphonate, pamidronate, bone metastases, prostate cancer, lutetium-177

## Abstract

Prostate cancer (PCa) is one of the most common cancer types worldwide. 90% of men with late stage PCa will develop bone metastases. Since the expression level of PSMA (prostate-specific membrane antigen) in bone metastases can vary significantly, a compound is being searched for which accumulates in bone metastases independently of PSMA level. With DOTA-L-Lys(SA.Pam)-PSMA-617, we present a compound that, in addition to a PSMA inhibitor as a target vector, also contains a bisphosphonate that is established as a bone tracer and thus combines the advantages of PSMA targeting and bone targeting. This is a class of small molecules combining targeting of two different targets with the potential advantages for treatment of biologically heterogeneous bone metastasis from prostate cancer. The molecule can be labeled with lutetium-177 and used for the therapy of PCa-related bone metastases. DOTA-L-Lys(SA.Pam)-PSMA-617 was synthesized and radiolabelled in 1 M ammonium acetate buffer pH 5.5 at 95°C. Different amounts of precursor were evaluated. Complex stability was evaluated in three different media. LogD_7.4_ value was evaluated via the determination of the equilibrium distribution in a PBS/n-octanol mixture. A hydroxyapatite binding assay was used to evaluate the potential binding to bone metastases. *In vitro* affinity was determined and K_i_ value was evaluated. To evaluate the binding potential in mice, *ex vivo* biodistribution studies were carried out in LNCaP tumor-bearing Balb/c mice. [^177^Lu]Lu-labeling of DOTA-L-Lys(SA.Pam)-PSMA-617 showed quantitative RCY within 10 min and high complex stability over 14 days. The lipophilicity of the labeled compound was similar to the lipophilicity of the reference compound [^177^Lu]Lu-PSMA-617 and showed an excellent and selective HAP binding of 98.2 ± 0.11%. With a K_i_ of 42.3 ± 7.7 nM PSMA binding affinity is lower in comparison to [^177^Lu]Lu-PSMA-617. First *ex vivo* biodistribution studies with LNCaP tumor-bearing Balb/c mice showed a PSMA dependent tumor accumulation of 4.2 ± 0.7%ID/g and a femur accumulation of 3.4 ± 0.4%ID/g. [^177^Lu]Lu-DOTA-L-Lys(SA.Pam)-PSMA-617 is a promising compound for therapy of PCa related bone and tissue metastases. Accumulation on the bone metastases *via* two mechanisms also enables the treatment of bone metastases that show little or no PSMA expression.

## Introduction

Prostate cancer (PCa) is one of the most commonly diagnosed cancer diseases in the world and one of the leading causes of cancer-related deaths worldwide (1). The 5-year survival rate for early diagnosed and localized PCa is 98%. However, if metastases have already formed, the rate decreases to 30% (2).

The introduction of prostate-specific membrane antigen (PSMA) as a molecular target has revolutionized the diagnosis and treatment of PCa. In recent years, there has been a rapid increase in new radiopharmaceuticals for diagnosis and targeted radionuclide therapy of PCa (3, 4). Particularly small molecule-based PSMA ligands have received a lot of attention (5, 6). The type II transmembrane glycoprotein PSMA or glutamate carboxypeptidase II (GCPII) consists of 750 amino acids and is located in the cell membrane of prostate epithelial cells. Usually, PSMA expression is upregulated in PCa and correlates with the aggressiveness of the disease (7). Apart from PCa, PSMA is physiologically expressed only in few normal tissues, such as the kidney and the salivary glands (8). Upon binding of a substrate to PSMA, internalization occurs. With regard to therapy, a targeted and irreversible uptake of the therapeutic nuclide into the PCa cell is thus possible (9).

The most commonly used PSMA inhibitors are based on urea derivatives. The currently most promising PET-PSMA radiopharmaceutical is PSMA-11, which can be labeled with gallium-68 (10). PSMA-617, labeled with lutetium-177 ([^177^Lu]Lu-PSMA-617, Lu-PSMA) is the most important therapeutic compound until now (8, 11, 12). The results of the clinical phase III study have just been published. The so-called VISION study demonstrated a good tolerability of the [^177^Lu]Lu-PSMA-617 therapy along with an increase in the overall survival rate. Hence, introduction of [^177^Lu]Lu-PSMA-617 as standard treatment is recommended (13). It is approved by the FDA since March 2022 (14).

Most men with advanced PCa develop bone metastases (15). This affects about 90% of men with late stage PCa (16). The presence of bone metastases is a negative indicator for the survival of the patient. The WARMTH study has shown that the overall survival rate of patients with bone metastases treated with [^177^Lu]Lu-PSMA-617 is lower than the overall survival rate of patients without bone metastases (17). Independent of this, bone metastases cause a significant reduction in quality of life due to, e.g., pain and pathological fractures or compression of the spinal cord and nerve roots or the displacement of red bone marrow, which appears especially in advanced PCa (18).

While metastasis in bone tissue can be osteoblastic, osteolytic or mixed forms, PCa-related metastases are usually osteoblastic (19, 20). In both cases, hydroxyapatite (HAP), the mineral material of the bone, is uncovered due to increased metabolism and bone remodeling (21, 22).

Over the last 40 years, bisphosphonates have become established as drugs for various bone diseases (23). The efficacy of these pyrophosphate derivatives is based on a high affinity to HAP, which is due to the chelation of the calcium ions of HAP (24). Bisphosphonates, such as MDP (methylene diphosphonate) or EDTMP (ethylenediamine tetra(methylene phosphonic acid)), are acyclic phosphonic acids that can both complex a radionuclide and adsorb to HAP, enabling diagnosis or therapy via the radioactivity of the nuclide (25). [^153^Sm]Sm-EDTMP is used, for example, for reduction of bone metastases-induced pain (26). In addition, there are bisphosphonates that are functionalised with a chelator that enables radiolabelling. These include compounds such as BPAMP (4-{[(bis-phosphonomethyl) carbomoyl]methyl}-7,10-bis-(carboxymethyl)-1,4,7,10-tetraazacyclododec-1-yl)-acetic acid) (27, 28) or the most promising [^177^Lu]Lu-DOTA^ZOL^ (DOTA-zoledronate) (29, 30).

One of the first clinically investigated nitrogen-containing bisphosphonate is pamidronate (Pam). The conjugation of Pam to squaric acid (SA) not only allows an easy and rapid conjugation with other moieties, such as chelators (31), but also supports the antiresorptive effect of the bisphosphonate due to the presence of an amine component at this position (32, 33). This is also the case, for example, with the highly potent bisphosphonate zolendronate (29, 30, 32). Unpublished data indicate that SA.Pam has a higher bone uptake than, for example, DOTA^ZOL^.

Prostate cancer can vary widely in the expression of PSMA. It is known that primary tumors and especially metastases can be PSMA-negative (34–38). This can also be the case for bone metastases (36, 37). Here, it was shown that there is a high correlation between bone metabolic activity and cancer-related PSMA expression in bone lesions at early stages of the disease, implying that bone metastases have significantly higher PSMA expression at early stages. In later stages, this correlation is increasingly absent or there is a greater deviation, which implies a lower PSMA expression in bone metastases (39). The reasons for this heterogeneous expression are manifold and many effects can play a role. These can be the complex biochemical and pathobiological processes due to genetically and non-genetically induced differentiation types of the tumor and the metastases in the progress of the disease (16, 37, 40, 41). Another influence can be, for example, the type of therapy (42).

It was found, that low average PSMA expression in metastases is associated with a shorter overall survival rate (41). At the moment, it is not yet clear whether patients with low PSMA-levels benefit from a therapy with lutetium-177 labeled PSMA-617 (41).

Therefore, there is a need for a compound that can accumulate at PCa-related bone metastases also independently of PSMA-level. In terms of imaging, different pairs of PSMA PET-tracers and bone scan agents have been studied in the past with varying results in their performance (39, 42–47). For example, a study by Rowe et al. showed that the PSMA inhibitor [^18^F]F-DCFPyl detected more bone lesions than the bisphosphonate [^99m^Tc]Tc-MDP and Na^18^F, which also have a high affinity toward HAP (44). In another study by Uprimmy et al. [^18^F]NaF outperformed [^68^Ga]Ga-PSMA-11 by finding more bone lesions (46).

The combination of the advantages of PSMA targeting and bone targeting in one compound would reduce the complexity of the treatment, enables additional expression-independent therapy of bone metastases and thus would be favorable. In this way, bone metastases can be treated *via* two mechanisms, the possible PSMA expression and the imbalanced metabolic mechanism of the bone cells. In this study, we developed a chimeric compound containing the highly affine PSMA inhibitor KuE on the one side and a bisphosphonate drug (pamidronate, Pam) on the other side. Both targeting moieties are coupled to the DOTA chelator and can therefore be used for endoradiotherapy with e.g., lutetium-177. Furthermore, we used the PSMA-617 linker for coupling the KuE unit to DOTA, since it has been proven that this linker plays an important role in enhancing the PSMA binding affinity (8). Moreover, pamidronate was coupled to DOTA via squaric acid amide, which has also shown a positive impact in terms of simplified synthesis and improved pharmacokinetics (48).

## Materials and Methods

### General

All chemicals were purchased from Sigma-Aldrich, Merck, Fluka, AlfaAesar, VWR, AcrosOrganics, TCI, Iris Biotech and Fisher Scientific and used without purification. Dry solvents were obtained from Merck and VWR, deuterated solvents for NMR spectra from Deutero. PSMA-617 was purchased from Hycultec. Thin layer chromatography was performed with silica gel 60 F254 coated aluminum plates from Merck. Evaluation was carried out by fluorescence extinction at λ = 254 nm and staining with potassium permanganate. The radio TLCs were evaluated using a CR-35 Bio test imager from Raytest and the AIDA (Raytest) software. The ^1^H and ^13^C NMR measurements were performed on an Avance III HD 300 spectrometer (300 MHz, 5 mm BBFO sample head with z-gradient and ATM and BACS 60 sample changer), an Avance II 400 spectrometer (400 MHz, 5 mm BBFO sample head with z-Gradient and ATM and SampleXPress 60 sample changer) and an Avance III 600 spectrometer (600 MHz, 5 mm TCI CryoProbe sample head with z-Gradient and ATM and SampleXPress Lite 16 sample changer) from Bruker. The LC/MS measurements were performed on an Agilent Technologies 1220 Infinity LC system coupled to an Agilent Technologies 6130B Single Quadrupole LC/MS system. Semi-preparative HPLC purification was performed on a 7,000 series Hitachi LaChrom and the respectively mentioned conditions and column. For radiolabelling experiments n.c.a. [^177^Lu]LuCl_3_ in 0.04 M HCl (ITM, Garching, Germany) was used.

### Organic Synthesis

#### Solid Phase Synthesis of the PSMA Ligand (PSMA-617-Resin)

The synthesis of the glutamate-urea-lysine binding motif and the linker of the PSMA-617 backbone was carried out following the established solid phase peptide chemistry as described by Benešová et al. (8, 11) with slight adjustments to the reaction procedures.

Bis(*tert*-butyl)-L-glutamate-hydrochloride (4.5 g, 15.21 mmol) and DIPEA (7.98 g, 10.5 mL, 61.74 mmol) were dissolved in dry dichloromethane (200mL) and cooled to 0°C. Triphosgene (1.56 g, 5.26 mmol) in dichloromethane (30 mL) were added dropwise over a period of 4.5 h. After the complete addition, the solution was stirred for an additional hour.

The Fmoc protecting group of Fmoc-L-Lysine(Alloc)-Wang resin (1.65 g, 1.5 mmol, 0.9 mmol/g) was removed by stirring it in a piperidine/DMF (1:1) solution for 15 min followed by a washing step with dichloromethane.

The deprotected L-lysine(Alloc)-Wang resin was added to the previous prepared solution and stirred over night at room temperature. The resin (compound **1**) was washed with dichloromethane (15 mL) and used without further purification.

Tetrakis(triphenylphosphin)palladium (516.0 mg, 0.45 mmol) und morpholine (3.92 g, 3.92 mL, 45.00 mmol) were dissolved in dichloromethane (12 mL) and added to compound **1**. The solution was stirred for 1 d under exclusion of light. Afterwards it was washed with dichloromethane (15 mL), a 1% DIPEA solution in DMF (3 x 13 mL) and a sodium diethyldithiocarbamat trihydrate solution (15 mg/mL) in DMF (9 x 10.5 mL x 5 min), resulting in compound **2**, the resin-immobilized and Alloc-deprotected glutamate-urea-lysine conjugate.

Fmoc-3-(2-naphthyl)-L-alanine (1.75 g, 4.00 mmol), HATU (1.52 g, 4.00 mmol), HOBt (540 mg, 4.00 mmol) and DIPEA (780 mg, 1.02 mL, 6.03 mmol) were dissolved in dry DMF (10 mL) and added to the resin. The solution was stirred overnight and then washed with DMF (10 mL) and dichloromethane (10 mL).

To remove the Fmoc-group, the resin (compound **3**) was stirred in a piperidine/DMF (1:1, 3 x 11 mL) solution for 10 min each and washed with DMF (10 mL) and dichloromethane (10 mL), resulting in compound **4**.

Fmoc-4-Amc-OH (1.52 g, 4 mmol), HATU (1.52 g, 4.00 mmol), HOBt (540 mg, 4.00 mmol) and DIPEA (780 mg, 1.02 mL, 6.03 mmol) were dissolved in dry DMF (10 mL) and added to the resin (compound **4**). The solution was stirred for 2 days and then washed with DMF (10 mL) and dichloromethane (10 mL).

To remove the Fmoc-group from compound **5**, it was stirred in a piperidine/DMF (1:1, 11 mL) solution for 10 min each and washed with DMF (10 mL) and dichloromethane (10 mL), leading to the final resin bound PSMA-617 backbone (**6**).

#### Pamidronate (7, Pam)

β-Alanine (1.5 g, 0.017 mol) and phosphorus acid (2.76 g, 0.034 mol) were dissolved in Sulfolane (5.5 mL) and cooled to 0 °C. Phosphorus trichloride (4.62 g, 2.95 mL, 0.034 mmol) was added dropwise. The solution was stirred for 3 h at 75 °C. Water (15 mL) was added and stirred for 12 h at 100°C. Ethanol (15 mL) was added and the product (**7**, 1.48 g, 0.006 mol, 37%) was obtained as yellow solid after crystallization at 0°C for 3 days.

^1^H-NMR (300 MHz, D_2_O) δ [ppm] = 3.34 (t, *J* = 7.1 Hz, 2H), 2.31 (tt, *J* = 13.7, 7.1 Hz, 2H).
^13^C-NMR (400 MHz, D2O): δ [ppm] = 72.58, 36.14, 30.54.
^31^P-NMR (121.5 MHz, D_2_O) δ [ppm] = 17.58 (s, 2P).
MS (ESI^+^): 236.0 [M+H]^+^, calculated for C_3_H_11_NO_7_P_2_: 235.07 [M]^+^.

#### Pamidronate-Squaric Acid Ethylester (8, SA.Pam)

Pamidronate (500 mg, 2.13 mmol) was dissolved in phosphate buffer (0.5 M, pH 7, 5 mL). 3,4-Diethoxycyclobut-3-ene-1,2-dione (squaric acid diethylester, SADE, 542 mg, 468 μL, 3.2 mmol) was added and the mixture was stirred for 2 days at room temperature. Ethanol (3mL) was added for crystallization. The mixture was allowed to stand for 3 days in the freezer to complete crystallization. The white precipitation was washed with cold ethanol and compound **8** (0.58 g, 1.62 mol, 76%) was obtained as white solid.

^1^H-NMR (400 MHz, D_2_O) δ [ppm] = 4.79-4.62 (m, 2H), 3.31 (t, *J* = 6.6 Hz, 2H), 2.32-2.15 (m, 2H) 1.42 (dt, *J* = 11.7, 7.2 Hz, 3H).
^31^P-NMR (162 MHz, D_2_O) δ [ppm] = 17,92 (s), 2.26 (s).
MS (ESI^+^): 360.0 [M+H]^+^,720.0 2[M+H]^+^, 763.0 2[M+Na]^+^, calculated for C_9_H_15_NO_10_P_2_: 359.16 [M]^+^.

#### Fmoc-L-Lys(Boc)-PSMA-617-Resin (9)

Fmoc-L-Lys(Boc)-OH (506 mg, 0.0011 mmol), HATU (415 mg, 0.0011 mg, HOBt (146 mg, 0.0011 mmol) and DIPEA (277 μL, 211 mg, 0.00162 mmol) were dissolved in acetonitrile (4 mL) and stirred for 30min. The PSMA-617-resin (300 mg, 0.0027 mmol, 0.09 mmol/g) was added and the mixture was stirred for 1 day at room temperature. The resin was washed with acetonitrile (10 mL) and dichloromethane (10 mL) and used in the next step.

#### L-Lys(Boc)-PSMA-617-Resin (10)

The Fmoc-L-Lys(Boc)-PSMA-617-resin (**9**) was stirred for 1 h in a mixture of DMF and Piperidine (1:1, 6 mL). The Fmoc deprotected resin was washed with DMF (10 mL) and dichloromethane (10 mL) and used in the next step without further purification.

#### DOTA(*t*Bu)_3_-L-Lys(Boc)-PSMA-617-Resin (11)

DOTA-Tris(*tert*-butyl ester) (310 mg, 0.54 μmol), HATU (308 mg, 0.00081 mmol), HOBt (110 mg, 0.00081 mmol) and DIPEA (184 μL, 140 mg, 0.0011 mmol) were dissolved in acetonitrile (4 mL) and stirred for 30 min. L-Lys(Boc)-PSMA-617-resin (**10**, 461 mg, 0.00027 mmol, 0.9 mmol/g) was added and the mixture was stirred for 1 day at room temperature. The resin was washed with acetonitrile (10 mL) and dichloromethane (10 mL) and used in the next step without further purification.

#### DOTA-L-Lys-PSMA-617 (12)

DOTA(*t*Bu)_3_-L-Lys(Boc)-PSMA-617-resin (**11**, 536 mg, 0.00027 mmol, 0.9 mmol/g) was stirred in a solution of TFA and dichloromethane (1:1, 4 mL) for 2 h. The TFA/dichloromethane solution was evaporated under reduced pressure and the product (**12**, 10.6mg, 0.0091 mmol, 4%) was obtained as a colorless powder after semi-preparative HPLC purification (column: LiChrospher 100 RP18 EC (250 x 10 mm) 5 μ, flow rate: 5 mL/min, H_2_O/MeCN + 0.1% TFA, 25% MeCN isocratic, t_R_ = 10.3 min).

MS (ESI^+^): 1172.5 [M+2H]^+^,585.9 1/2[M+2H]^+^, 391.0 1/3[M+2H]^+^, calculated for C_55_H_83_N_11_O_17_: 1170.33 [M]^+^.

#### DOTA-L-Lys(SA.Pam)-PSMA-617 (13)

Compound **12** (10 mg, 0.0085 mmol) and compound **8** (16 mg, 0.043 mmol) were dissolved in phosphate buffer (0.5 M, pH 9, 1mL) and stirred for 2 days. The product (**13**, 10.56 mg, 0.0071 mmol, 84%) was obtained as a colorless powder after semi-preparative HPLC purification (column: LiChrospher 100 RP18 EC (250 x 10 mm) 5μ, flow rate: 5 mL/min, H_2_O/MeCN + 0.1% TFA, 23% to 28% MeCN in 20 min, t_R_ = 8.2 min).

MS (ESI^+^): 511.3 1/3[M+H+2Na]^+^, 520.0 [1/3M+2K]^+^, 781.0 1/2[M+2K]^+^, calculated for C_62_H_92_N_12_O_26_P_2_: 1483.42 [M]^+^.

### Radiolabelling of DOTA-L-Lys(SA.Pam)-PSMA-617 With Lutetium-177 ([^177^Lu]Lu-13)

For radiolabelling experiments n.c.a. [^177^Lu]LuCl_3_ in 0.04 M HCl (ITG, Garching, Germany) was used.

Radiolabelling was performed in 1 mL 1 M ammonium acetate buffer at pH 5.5. Reactions were carried out with different amounts of precursor (5, 10, 30 nmol) and at 95 °C with 40–50 MBq n.c.a. lutetium-177. Radio-TLC (TLC Silica gel 60 F254 Merck) and citrate buffer (pH 4) as mobile phase and radio-HPLC using an analytical HPLC 7000 series Hitachi LaChrom (Column: Merck Chromolith® RP-18e, 5-95% MeCN (+0,1% TFA)/ 95-5% Water (+0,1% TFA) in 10 min) were used for reaction control. TLC's were measured in TLC imager CR-35 Bio Test-Imager from Elysia-Raytest (Straubenhardt, Germany) with AIDA software.

### *In vitro* Stability Studies

Complex stability studies were carried out in human serum (HS, human male AB plasma, USA origin, Sigma Aldrich), NaCl and phosphate buffered saline (Sigma Aldrich). 5 MBq of the radioactive compound were incubated in 0.5 mL of the media for 14 d. After several time points (1, 2, 5h, 1, 2, 5, 7, 9, and 14d), aliquots were taken out to evaluate the radiochemical stability. Experiments were carried out as triplicate.

### Lipophilicity Determination

Using the shake flask method, the logD_7.4_ value of the compound was determined. The labeling solution was adjusted to pH 7.4 and 5 MBq were diluted in 700 μL n-octanol and 700 μL PBS. It was shaken for 2min at 1500 U/min and then centrifuged. 400 μL of the n-octanol phase and 400 μL of the PBS phase were each transferred to a new Eppendorf tube. 3-6 μL were then pipetted on a TLC plate and analyzed via phosphor imager. LogD_7.4_ value was calculated by determining the ratio of the activities of the two phases. This procedure was repeated twice more with the respective phase with higher activity, so that three coefficients were obtained and the mean value was calculated.

### Binding Studies of ^177^Lu-Labeled Compound on Hydroxyapatite

Hydroxyapatite (20 mg) was incubated in saline (1 mL) for 24 h. 50 μL of the labeled [^177^Lu]Lu-**13** (5 MBq) and respectively [^177^Lu]Lu-PSMA-617 (5 MBq) was added. The suspension was vortexed for 20 s and incubated for 1 h at room temperature. The samples were passed through a filter (CHROMAFIL® Xtra PTFE-45/13) and the supernatant was washed with water (500 μL). The radioactivity of the liquids and the HAP-containing supernatant were measured with a curiemeter (Aktivimeter Isomed 2010 MED Nuklear-Medizintechnik Dresden GmbH). The binding of [^177^Lu]Lu-**13** and [^177^Lu]Lu-PSMA-617 was determined as percent of activity absorbed to HAP. Free lutetium-177 was examined as a positive probe analogously.

For blocking experiments, Hydroxyapatite (20 mg) was incubated in saline (1 mL) with pamidronate (100 mg) and evaluated with [^177^Lu]Lu-**13** and free lutetium-177 as described above.

### *In vitro* Binding Affinity

Cold [^nat^Lu]Lu complexes were synthesized by shaking a mixture of **13** (371 μL of a 1 mg/ml solution, 250 nmol) and LuCl_3_ (129 μL of a 1 mg /mL solution, 375 nmol; metal to ligand ratio 1.5 to 1) in 1 M ammonium acetate buffer at 95 °C for 2 h. Complexation was monitored by ESI-LC/MS.

Based on the protocol published by Benešová et al. (8) the PSMA binding affinity was determined in a competitive radioligand assay. PSMA-positive LNCaP-cells (purchased from Sigma-Aldrich) were cultured in RPMI 1640 (Thermo Fisher Scientific) supplemented with 10% fetal bovine serum (Thermo Fisher Scientific), 100 μg/ml streptomycin, and 100 units/ml penicillin at 37°C in 5% CO_2_. These cells were incubated for 45 min with rising concentrations of the test compounds in the presence of 0.75 nM [^68^Ga]Ga-PSMA-10. Free radioactivity was removed by several washing steps with ice-cold PBS. Probes were measured in a γ-counter (2480 WIZARD^2^ Automatic Gamma Counter, PerkinElmer). Obtained data were analyzed in GraphPad Prism 9 using non-linear regression.

### Animal Studies

All animal experiments were approved by the ethical committee of the state of Rhineland Palatinate (according to §8 Abs. 1 Tierschutzgesetz, Landesuntersuchungsamt) and performed in accordance with relevant federal laws and institutional guide-lines. Approval Nr. 23 177-07/G 21-1-022.

6- to 8-week-old male BALB/cAnNRj (Janvier Labs) were inoculated subcutaneously with 5x10^6^ LNCaP cells in 200 μL 1:1 (v/v) Matrigel/PBS (Corning®). Experiments are conducted after the tumor has reached a volume of approximately 100 cm^3^.

LNCaP xenografts were anesthetized with 2% isoflurane prior to i.v. injection of 0.5 nmol of [^177^Lu]Lu-**13**. The specific activity was approximately 3 MBq/nmol. PSMA-selectivity was investigated by co-injection of 1.5 μmol PMPA/mouse.

Animals were sacrificed 24 h p.i. Organs were collected and weighed. The radioactivity was measured and calculated as decay-corrected percentage of the injected dose per gram of tissue mass %ID/g.

## Results

### Organic Synthesis of DOTA-L-Lys(SA.Pam)-PSMA-617

**Figure 1:**
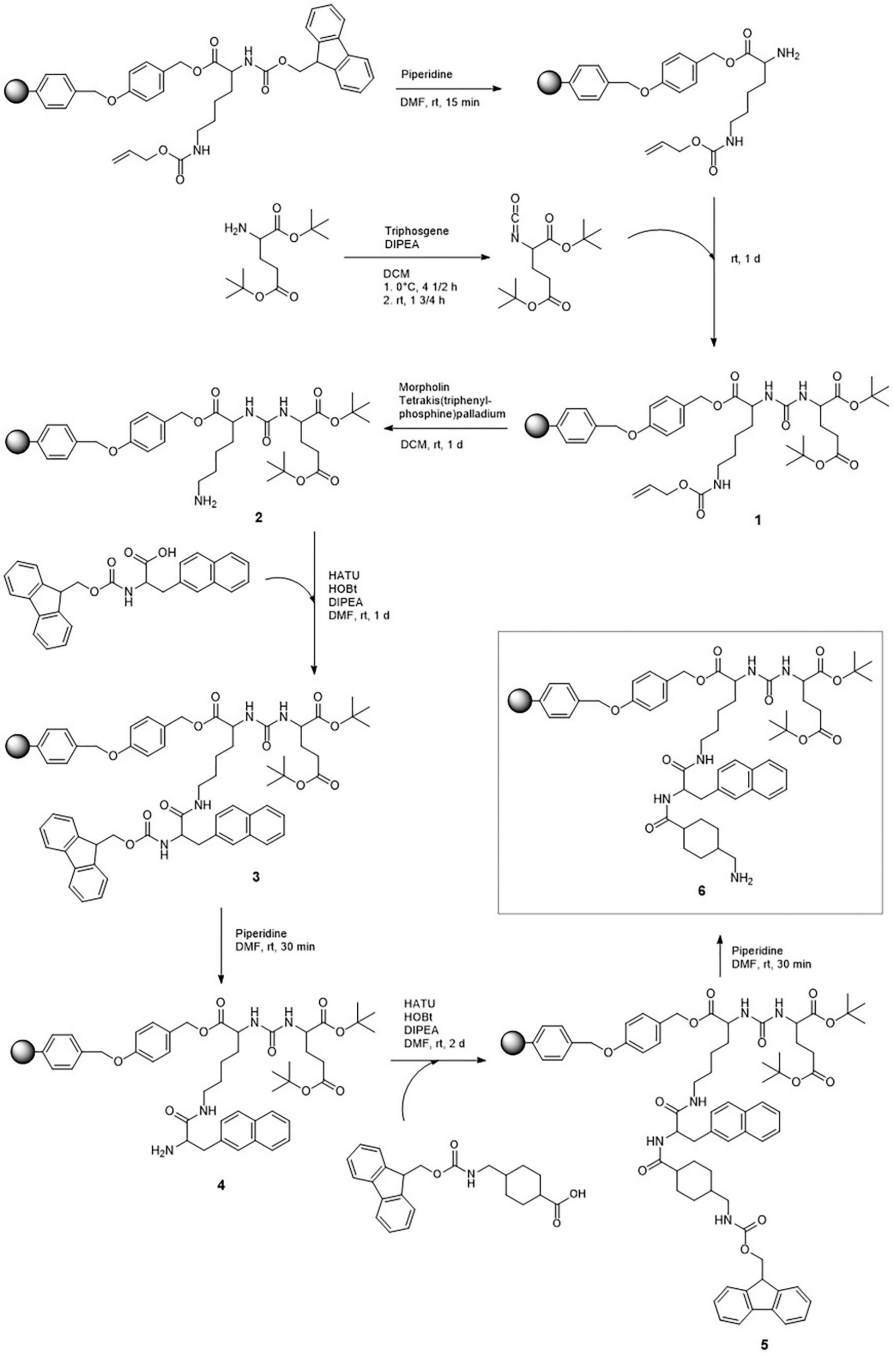
Synthesis scheme of the resin bound PSMA-617 backbone.

**Figure 2:**
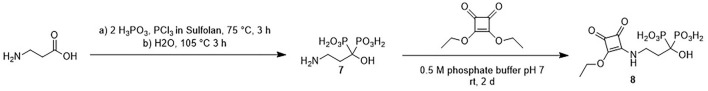
Synthesis scheme of the pamidronate target vector **8**.

**Figure 3:**
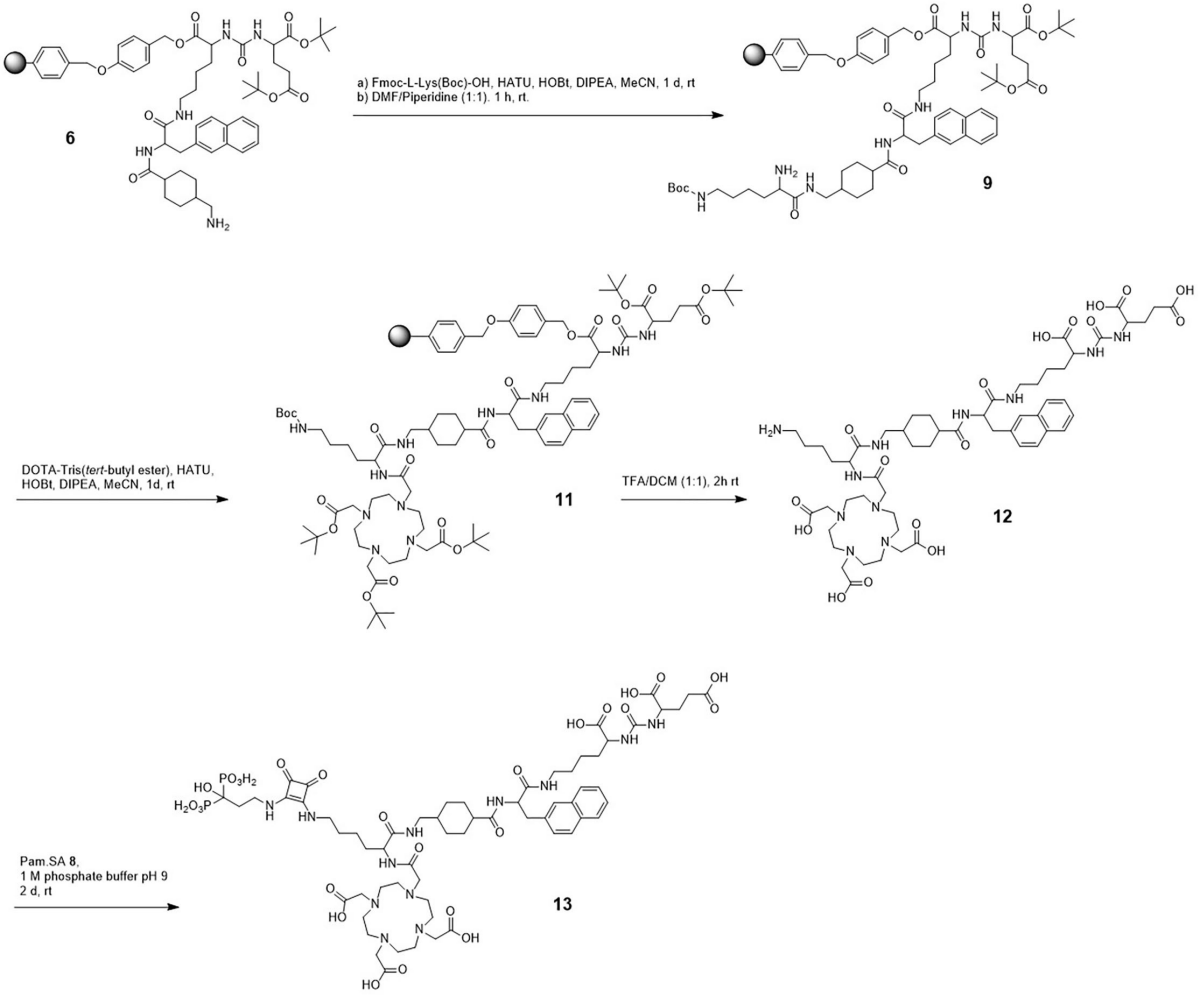
Synthesis scheme of DOTA-L-Lys(SA.Pam)-PSMA-617 **13**.

The synthesis of DOTA-L-Lys(SA.Pam)-PSMA-617 was divided into three parts. First the solid-phase based synthesis route of the PSMA-617 motif (**6**) based on the synthetic route developed by Benešová et al. (8, 11) (Figure 1). The bisphosphonate-based target vector (**8**) unit was synthesized in a two-step synthesis (Figure 2). Both target vectors were then combined in a third synthesis step using lysine as a linking bridge and functionalised with a DOTA chelator for radiolabelling (Figure 3).

The eight-step solid-phase synthesis of the PSMA-617-backbone (Figure 1) started from L-lysine which is bound to a Wang-resin. In the first step, an isocyanate was generated by adding triphosgene to bis(*tert-*butyl)-L-glutamate, which was then added to the Fmoc-deprotected lysine-resin to generate the KuE unit of the PSMA inhibitor. To add the PSMA-617-linker unit, the alloc protecting group of the side chain amine of **1** was removed by reduction. This was followed by addition of the amino acids 3-(2-napthyl)-L-alanine and 4-aminomethylcyclohecxane-OH (4-Amc-OH) to the side chain amine of lysine, using a standardized solid-phase based peptide synthesis protocol with HATU (1-[Bis(dimethylamino)methylene]-1H-1,2,3-triazolo[4,5-b]pyridinium 3-oxide hexafluorophosphate) as coupling reagent. The final solid-phase bound and protected PSMA-617-target vector motif (**5**) was obtained. The Fmoc protecting group of the aminomethylcyclohexane group was removed for further functionalisation.

The second target vector, the squaric acid conjugated bisphosphonate pamidronate (**8**) was synthesized in a two-step synthesis with an overall yield of 28% (Figure 2). According to literature, β-alanine was transformed into pamidronate (**7**) using phosphorus acid and phosphorus trichloride (49). This was followed by an asymmetric amidation to conjugate the amine of the bisphosphonate to the squaric acid ester.

The final part of the synthesis was the conjugation of the two target vectors **6** and **8** and the DOTA-chelator using lysine as bridging unit (Figure 3). First, protected lysine was conjugated to the free amine of the resin bound compound **6**. After Fmoc deprotection, DOTA-Tris(*tert*-butyl ester) was conjugated. Both steps were carried out with HATU as reagent for amide formation. This reaction led to the complete protected and resin bound compound **11**. This compound was completely deprotected under acidic conditions and removed from the solid phase. In the further process of the reaction, protective groups are no longer necessary, as the following asymmetric amidation is highly selective for amines. Compound **12** was purified via semi preparative HPLC resulting in a yield of 4% after the amide formation steps. Finally, the second target vector Pam.SA (**8**) was added-this was done via the asymmetric amidation of the squaric acid monoester and the free amine of compound **12**. Due to poor solubility of the bisphosphonate **8** in organic solvents, this reaction was carried out in aqueous phosphate buffer at a pH value of 9. The final compound **13** was purified via semi-preparative HPLC with a yield of 84%. The entire compound DOTA-L-Lys(SA.Pam)-PSMA-617 was prepared in 15 steps with a total yield of 1%.

### Radiochemical Evaluation with Lutetium-177

**Figure 4:**
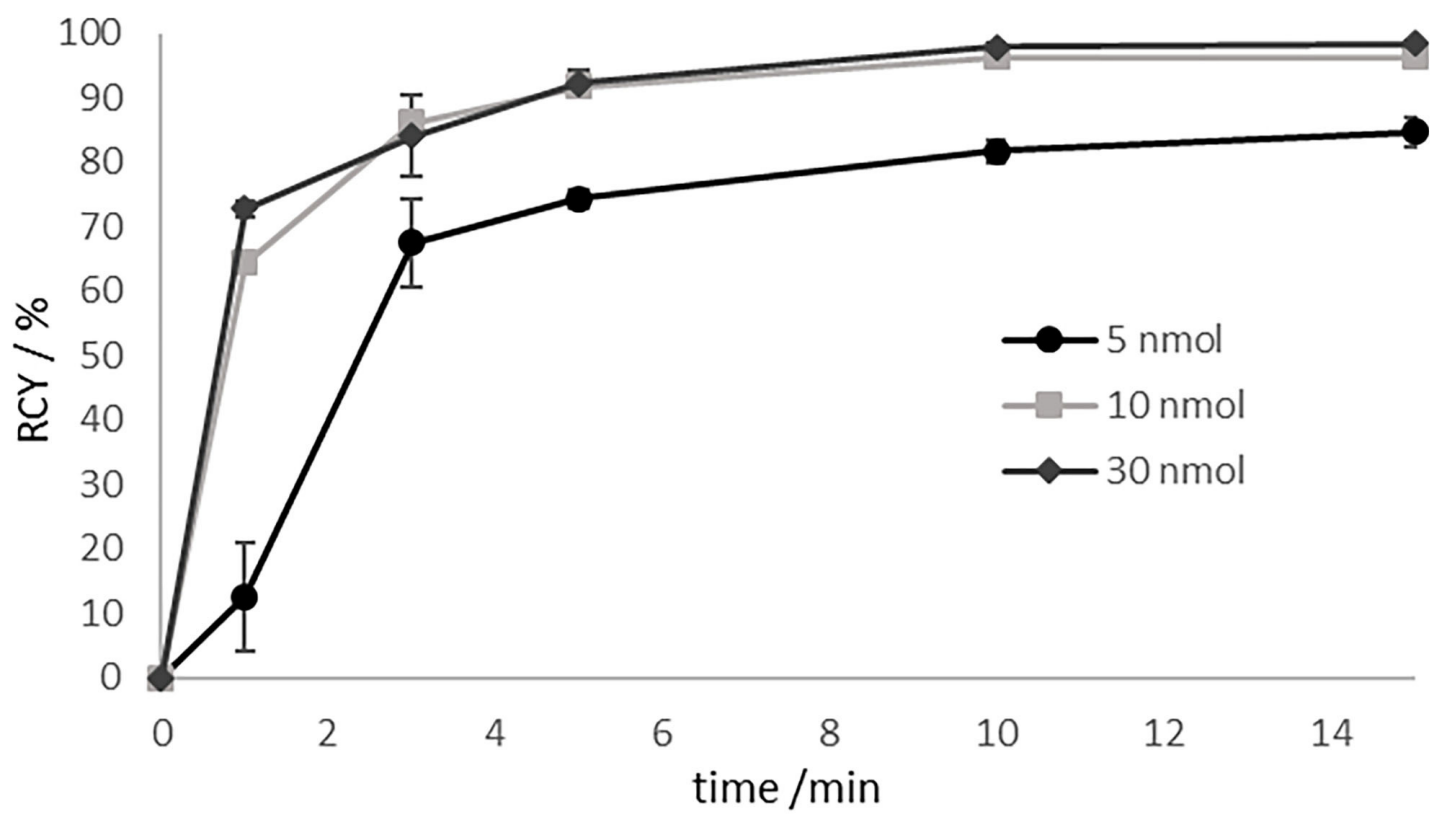
Labeling kinetics of [^177^Lu]Lu-**13** for various amounts of precursor at 95 °C in 1 M ammonium acetate buffer pH 5.5 at 95°C. Precoursor amounts of >10 nmol resulting in RCYs of > 96% after 10 min.

Radiolabelling of compound **13** with lutetium-177 was carried out at 95°C in 1 mL of ammonium acetate buffer (1 M, pH 5.5). The radiochemical yield (RCY) as a function of precursor amount (5 to 30 nmol) was evaluated and illustrated in Figure 4.

**Figure 5:**
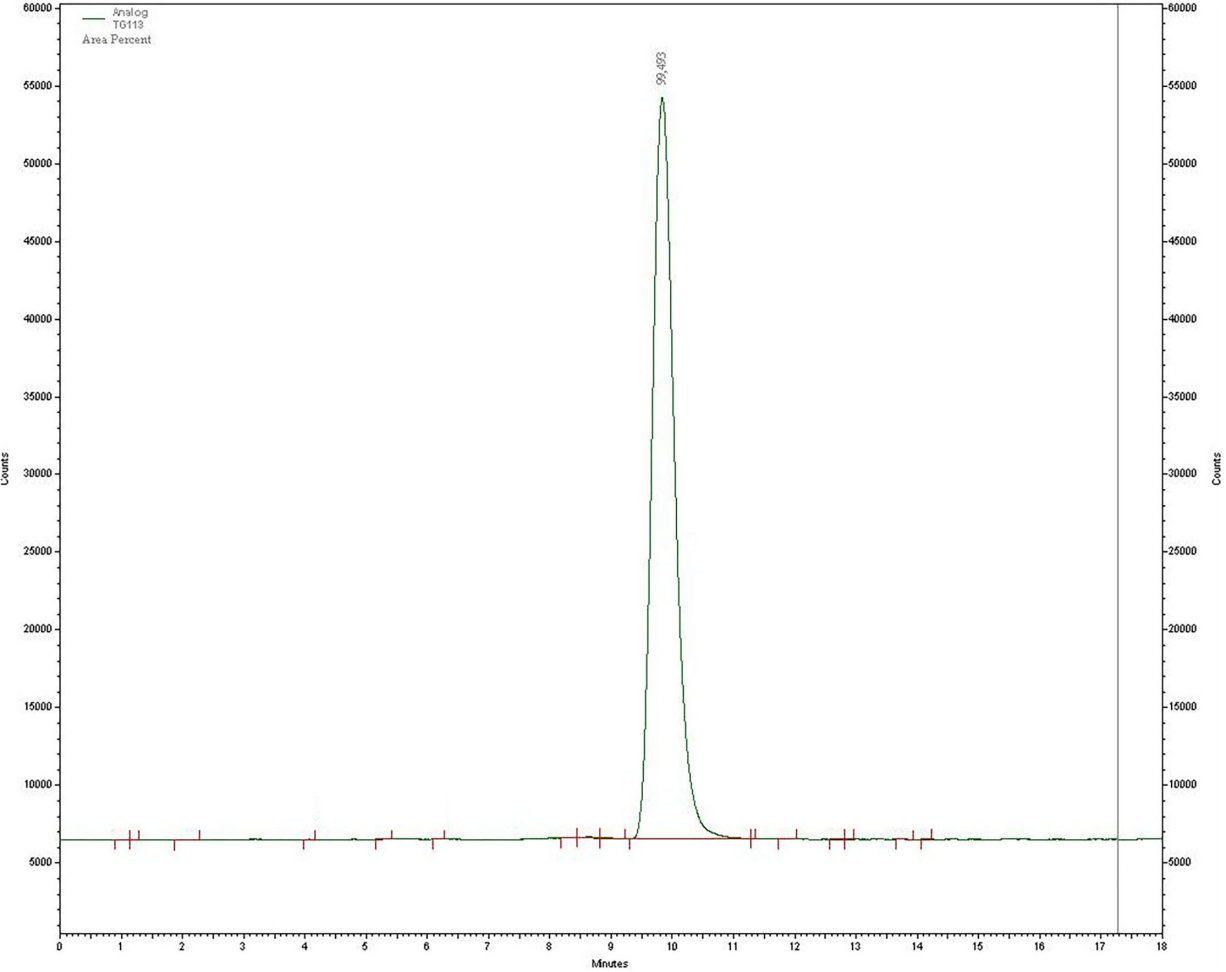
HPLC chromatogram after labeling of **13** with lutetium-177 (t_R_ = of 9.8 min).

For substance amounts higher than 10 nmol, radiochemical yields of over 90% were already achieved after 5 min. The RCY was almost quantitative after 10 min. In contrast, with a substance quantity of 5 nmol, the RCY obtained after 5 min was of only 75%. In the further progress of the reaction, the yield increased to 85%, but then stagnated there indicating that the labeling kinetics are depending on the amount of the precursor. RCY and radiochemical purity were analyzed by radio-TLC and radio-HPLC. On radio-TLC the labeled compound [^177^Lu]Lu-**13** showed a R_f_ of 0.0, while free unlabelled [^177^Lu]Lu^3+^ showed a R_f_ of 0.8–1 in citrate buffer as mobile phase. On analytical radio-HPLC, the compound had a retention time (t_R_) of 9.8 min (see Figure 5).

**Figure 6:**
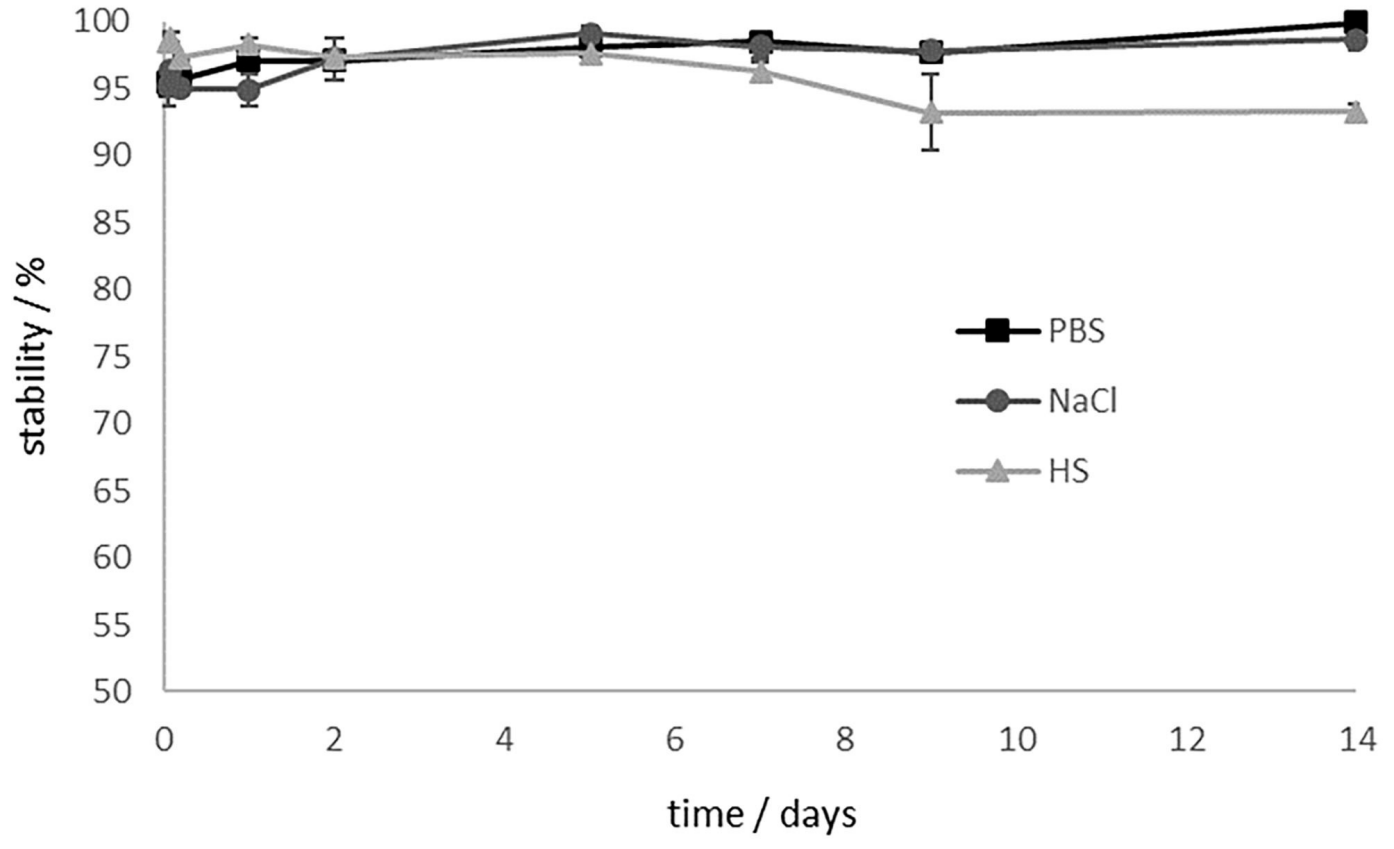
Stability studies of [^177^Lu]Lu-13 in different media for 14 d. [^177^Lu]Lu-**13** is stable in PBS and NaCl. The stability in HS decreases to 93% after 9 days.

Since the stability of the ligand-chelator conjugate is crucial for a translational use, complex stability studies were carried out in different media (phosphate buffered saline (PBS), isotonic saline (NaCl) and human serum (HS)). Results are shown in Figure 6.

In PBS and saline, [^177^Lu]Lu-**13** showed > 98% intact conjugate even after 14 days. Stability in human serum decreases slightly. However, after 9 d, 93% are still intact. Stability is preserved and even after 14 days the stability in HS is still at 93%.

The lipophilicity of the compound was determined via the equilibrium distribution in a mixture of n-octanol and PBS using the shake flask method. LogD_7.4_ values of [^177^Lu]Lu-**13** and the reference compound [^177^Lu]Lu-PSMA-617 are shown in Table 1. The lipophilicity of the lutetium-labeled compound **13** was similar to the lipophilicity of [^177^Lu]Lu-PSMA-617.

**Table 1:** Experimentally determined logD_7.4_ values of [^177^Lu]Lu-**13** and the reference [^177^Lu]Lu-PSMA-617.

**Compound**	**logD_**7.4**_ (n-octanol/PBS)**
[^177^Lu]Lu-**13**	−2.29 ± 0.12
[^177^Lu]Lu-PSMA-617	−2.23 ± 0.20

### Binding Studies on Hydroxyapatite (HAP)

**Figure 7:**
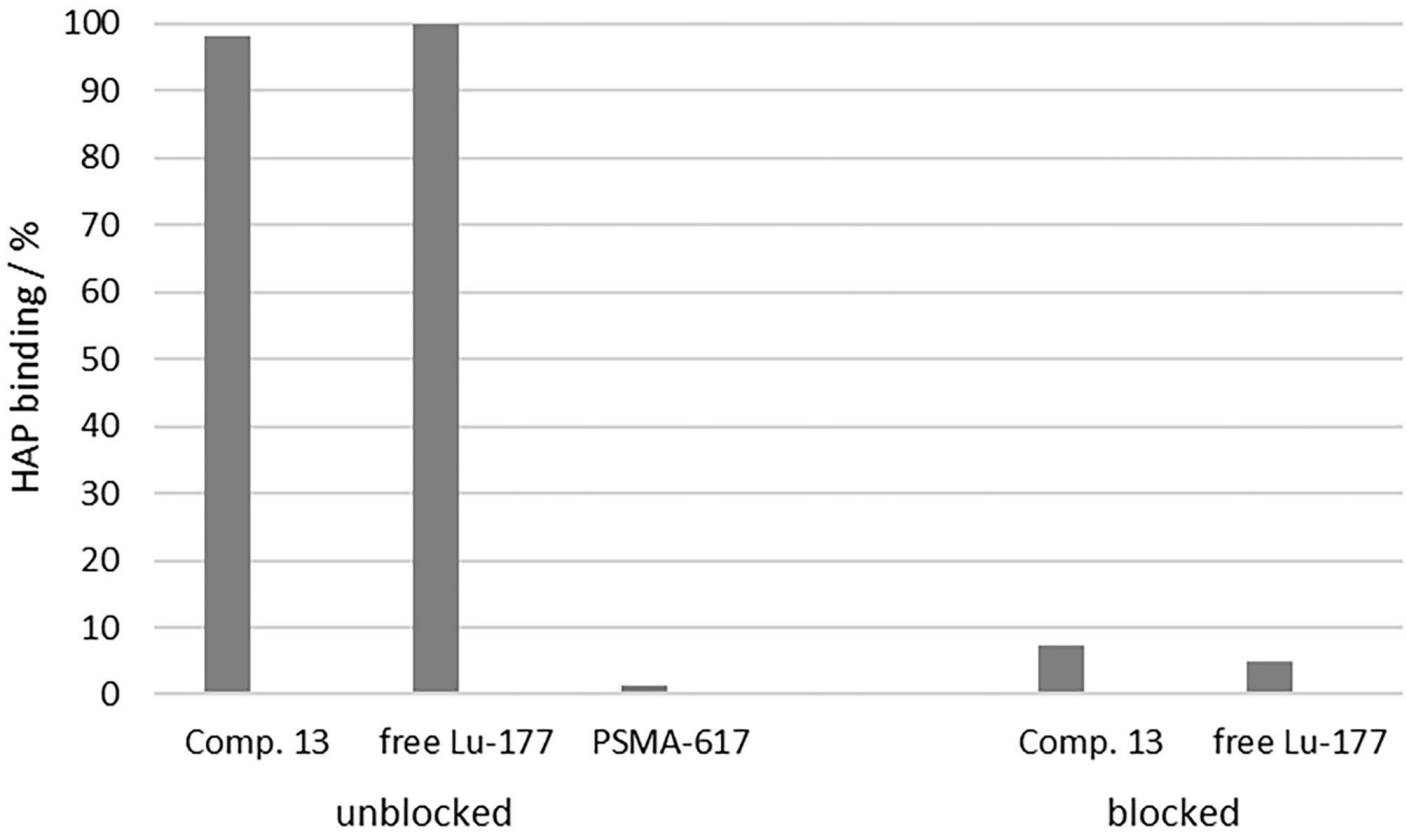
Percent enrichment of [^177^Lu]Lu-13 (98.2 ± 0.11%), [^177^Lu]Lu-PSMA-617 (1.20 ± 0.30%) and free [^177^Lu]Lu^3+^ (99.89 ± 0.02%) to HAP in comparison to the percent enrichment of [^177^Lu]Lu-**13** (7.31 ± 1.08%) and free [^177^Lu]Lu^3+^ (4.89 ± 0.51%) to HAP previously blocked with pamidronate.

The Ca-containing hydroxyapatite is found in mammal bones (50). Crystalline HAP is therefore suitable as a model compound to investigate the accumulation potential of bisphosphonates to the bone structure or bone metastases *in vitro*. Figure 7 shows the enrichment of [^177^Lu]Lu-**13**, [^177^Lu]Lu-PSMA-617 and free [^177^Lu]Lu to HAP in comparison to the enrichment of [^177^Lu]Lu-**13** and free [^177^Lu]Lu^3+^ to HAP previously blocked with pamidronate.

Uncomplexed lutetium cation [^177^Lu]Lu^3+^ is known for its high affinity toward HAP (50). Therefore, it was used as positive sample. In this study, it showed a HAP-binding of 99.89 ± 0.02%. [^177^Lu]Lu-**13** also showed also a nearly complete enrichment at the HAP surface (98.2 ± 0.11%), comparable to the free lutetium cation. In contrast, [^177^Lu]Lu-PSMA-617 displayed no notable binding (1.20 ± 0.30%). To evaluate the selectivity of binding, HAP was blocked with an excess of pamidronate prior to incubation with the test compounds. The binding of [^177^Lu]Lu-**13** (7.31 ± 1.08%) as well as [^177^Lu]Lu^3+^ (4.89 ± 0.51%) decreased significantly, indicating a specific binding to the calcium ions of the apatite structure.

### *In vitro* Affinity Assay

In a further step, we evaluated the PSMA binding affinity of compound **13** in a competitive radioligand assay and compared the calculated K_i_ value to the inhibition constant values of compound **12** to evaluate the influence of the SA.Pam unit and to PSMA-617 as seen in Table 2. Compound **13** showed good binding affinity similar to that of [^nat^Lu]Lu-**13**, thus indicating that radionuclide complexation does not have any impact on PSMA-binding. However, the bisphosphonate-free PSMA tracer DOTA-L-Lys-PSMA-617 (compound **12**) displayed 2-fold higher binding potency than the pamidronate-PSMA conjugate, indicating an influence of the SA.Pam unit. In addition, the binding affinity of the reference compound PSMA-617 in this assay is also higher than in comparison to compound **13**. Compound **12** shows a 2.5-fold lower affinity in contrast to PSMA-617.

**Table 2:** Inhibition constant values of PSMA ligands.

**Compound**	***K_***i***_ [nM]***
Compound **13**	52.6 ± 3.5
[^nat^Lu]Lu-**13**	42.3 ± 7.7
Compound **12** (DOTA-L-Lys-PSMA-617)	19.7 ± 3.3
PSMA-617^*^	6.9 ± 1.3

* Values are mean ± SD. ^*^IC_50_ values can be found at Greifenstein et al. (48).

### *Ex vivo* Studies

**Figure 8:**
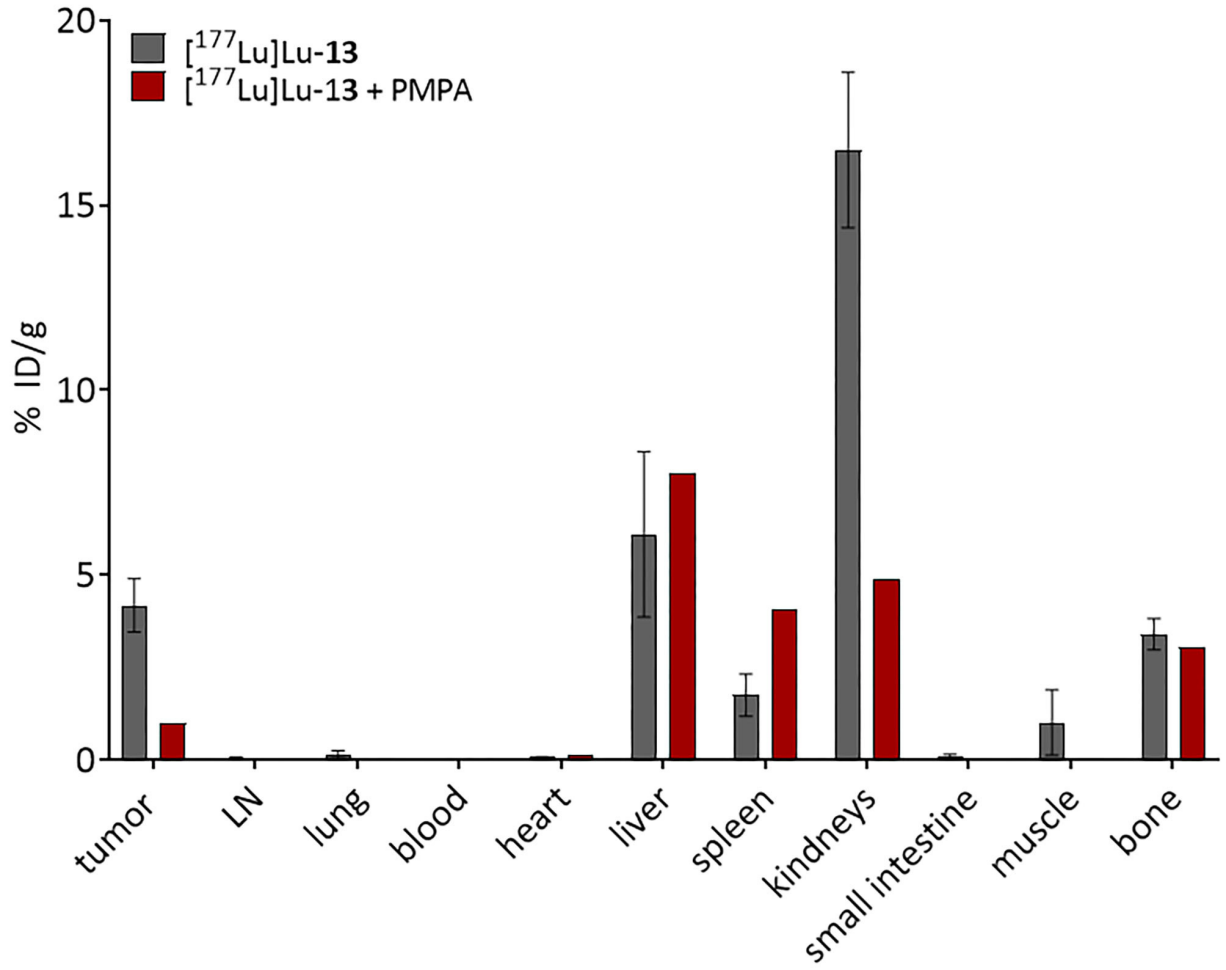
Biodistribution of 0.5 nmol [^177^Lu]Lu-**13** at 24 h p.i. (*n* = 3). PSMA-selectivity was assessed by co-injection of 1,5 mol PMPA. Data are % injected dose per gram tissue. The figure was created with GraphPad Prism version 9, GraphPad Software, San Diego, California USA, www.graphpad.com.

Biodistribution studies of [^177^Lu]Lu-**13** were performed using LNCaP tumor-bearing Balb/c mice. The accumulation of the ^177^Lu-labeled tracer in several organs was determined and illustrated in Figure 8. Although the accumulation of the tracer in both tumor and femur were similar (4.2 ± 0.7 and 3.4 ± 0.4%ID/g, respectively), the bone-uptake was, in contrast to tumor-uptake, not PSMA-specific since it could not be blocked by the PSMA inhibitor PMPA (2-(Phosphonomethyl)pentanedioic acid). Additionally, [^177^Lu]Lu-**13** showed the highest accumulation in the kidneys 16.5 ± 2.1%ID/g, which seems to be PSMA-specific because it could be reduced by co-injection of PMPA. In contrast, the uptake in the liver as well as in the spleen was PSMA-unspecific. Noteworthy is the high tumor-to-blood and bone-to-blood ratios (210 and 170, respectively) resulting from a minimal accumulation in the blood.

## Discussion

Due to the heterogeneity in PSMA-expression of many bone metastases, the dual-targeting strategy could be an advantageous approach in the treatment of prostate cancer-related bone metastases. A new heterodimeric compound containing a PSMA inhibitor target vector, a bisphosphonate drug and a DOTA chelator was developed and evaluated regarding radiolabelling, lipophilicity, HAP binding, *in vitro* affinity toward PSMA and *ex vivo* kinetic properties.

The compound DOTA-L-Lys(SA.Pam)-PSMA-617 (**13**) was prepared in 15 steps with a total yield of 1%.

Radiolabelling of **13** with lutetium-177 showed a fast and quantitative RCY after 10 min with quantities over 10 nmol. The ^177^Lu-labeled compound is stable (<98%) in PBS and saline for 14 days and in HS for 5 days, releasing 7% of the lutetium-177 until day 9 and remaining stable even after 14 days (93%). Thus, the complexation of the therapeutic nuclide seems to be stable even after 2 weeks. Due to the long half-life of lutetium-177 (7 days), it is also possible to store the labeled compound for a short time before injection, for example, for the treatment of several patients with one batch of tracer. The most suitable medium here is isotonic saline, which could also serve as injection medium. Moreover, the high stability of [^177^Lu]Lu-**13** in HS makes it unlikely that lutetium-177 will be released during the circulation time in the blood. The experimentally determined logD_7.4_ value as a measure of lipophilicity is almost identical to the value of [^177^Lu]Lu-PSMA-617, which serves as reference, indicating the hydrophilic character of both compounds.

An aqueous suspension of crystalline HAP was used as a bone tissue model to test the HAP binding potency of the novel compound. The adsorption of [^177^Lu]Lu-**13** to HAP was almost quantitative. It is nearly identical to the adsorption shown by free [^177^Lu]Lu^3+^. Uncomplexed [^177^Lu]Lu^3+^ is known for its high HAP affinity and correspondingly strong accumulation in bone tissue (51). Compared to other literature-known DOTA conjugated bisphosphonates, e.g., [^68^Ga]Ga-DOTA^PAM^ (DOTA-Pamidronate; 91.2 ± 2.7%), [^68^Ga]Ga-DOTA^ZOL^ (92.7 ± 1.3%) or [^68^Ga]Ga-BPAPD (4-{[(bis-phosphonopropyl)carbomoyl]methyl}7,10-bis-(carboxymethyl)-1,4,7,10-tetraazacyclododec-1-yl)-acetic acid) (83.0 ± 0.8%), [^177^Lu]Lu-**13** showed an even better adsorption (52).

By blocking of HAP with an excess of pamidronate prior to addition of [^177^Lu]Lu-**13** or [^177^Lu]Lu^3+^, no significant adsorption was observed. This proves that the selective binding of [^177^Lu]Lu-**13** to HAP is due to the adsorption of the SA.Pam unit at the calcified HAP surface. As expected, [^177^Lu]Lu-PSMA-617 showed no notable binding to HAP. This shows that, in contrast to [^177^Lu]Lu-**13**, [^177^Lu]Lu-PSMA-617 can only accumulate in bone metastases via one mechanism, namely PSMA-binding.

In addition to HAP adsorption, [^nat^Lu]Lu-**13** displayed a PSMA binding affinity in the nanomolar range. The complexation of the radiometal had no impact on the binding potency, since the K_i_ values of both complexed and uncomplexed conjugates were similar (42.3 ± 7.7 nM and 52.6 ± 3.5 nM for [^nat^Lu]Lu-**13** and comp **13**, respectively) (52). However, the bisphosphonate-free compound **12** showed 2-fold higher binding potency revealing that the coupling of pamidronate affects the interaction of the tracer with the PSMA binding pocket, possibly through changes in the conformation of the molecule.

Biodistribution studies were conducted in LNCaP tumor-bearing mice in order to better characterize the translational potential of the dual-targeting properties of [^177^Lu]Lu-**13**. In terms of PSMA-specific accumulation, [^177^Lu]Lu-**13** showed good PSMA-specific tumor accumulation 4.2 ± %ID/g which could be reduced by blocking the PSMA receptors with the potent PSMA inhibitor PMPA. According to literature the tumor-uptake of [^177^Lu]Lu-PSMA-617 seems to be significantly higher (11.2 ± 4.17%ID/g) (8). However, this could be due to the higher binding affinity of this tracer (K_i_ = 6.9 ± 1.3 vs. 42.3 ± 7.7 nM for [^nat^Lu]Lu-**13**). Nevertheless, [^177^Lu]Lu-PSMA-617 did not display any binding to HAP as discussed above, thus accumulation in PSMA negative bone metastases is supposed to be minimal since bone-uptake is depending on PSMA-expression. In contrast, [^177^Lu]Lu-**13** showed bone accumulation in the same range as tumor accumulation. This could not be blocked by PMPA suggesting that bone uptake is solely resulting from HAP binding. As already discussed, compound **13** can therefore be used in contrast to PSMA-617 for the therapy of PSMA negative bone metastases, since it can also accumulate at the metastases via the increased bone metabolism. Compared to the results published by Meckel et al. (52) [^177^Lu]Lu-**13** showed similar bone uptake as [^177^Lu]Lu-DOTA^ZOL^ (3.4 ± 0.4 and 3.2 ± 0.4%ID/g, respectively). However, kidney accumulation of [^177^Lu]Lu-**13** was higher than [^177^Lu]Lu-DOTA^ZOL^ (16.5 ± 2.1 vs. 1,3 ± 0.1%ID/g, respectively). This could be due to the existence of PSMA receptors in the kidneys as known from literature (37). Furthermore, the uptake in the liver and the spleen seems to be PSMA-independent, since no blocking effects were observed. The reason has yet to be found, as increased liver uptake is not known here for either bisphosphonates or PSMA-617. Nevertheless, target-to-background ratio of [^177^Lu]Lu-**13**, 24h after injection, was remarkably high (tumor-to-blood-ratio, 210; bone-to-blood ratio, 170) revealing the promising potential of this compound. Especially with regard to the conjugated DOTA-chelator, which is a versatile chelator not only used in complexing the therapeutic β^−^-nuclide lutetium-177, which is evaluated in this study, but is also applicable with other clinically relevant nuclides used in diagnosis by PET (e.g., ^68^Ga or ^44^Sc) or SPECT (e.g., ^111^In or ^67^Ga). Furthermore, the use of compound **13** in combination with α-emitters (e.g., ^225^Ac or ^213^Bi) would be interesting for the treatment of bone metastases in close distance to the bone marrow due to the short range of these nuclides.

## Conclusion

In this work, a novel chimeric compound for therapy of prostate cancer related bone metastases was designed and successfully synthesized. Key structure elements of the novel compound are the following. First, the well-known and established PSMA inhibitor KuE in combination with the PSMA-617 linker unit enables high affinity toward PSMA. Second, the bisphosphonate pamidronate as HAP target vector shows high bone accumulation. It is conjugated via SA unit, which allows easy conjugation chemistry as well as increase of the antiresorptive effect of the bisphosphonate. Finally, the DOTA chelator allows the labeling with a wide range of diagnostic and therapeutic radiometals.

DOTA-L-Lys(SA.Pam)-PSMA-617 shows both excellent labeling properties and very good and selective HAP binding, which is superior to other DOTA-conjugated bisphosphonate compounds. The *in vitro* PSMA affinity is lower than that of PSMA-617, which can be ascribed to an influence of the Pam.SA group. Nevertheless, the compound shows both PSMA-dependent tumor uptake and PSMA-independent and pamidronate driven uptake into bone tissue and a remarkably high tumor-to-blood as well as bone-to-blood-ratio after 24 h.

The available results show that the new compound may be suited for the therapy of PCa-related metastases in soft tissue as well as bone. Despite the lower affinity and *in vivo* uptake compared to [^177^Lu]Lu-PSMA-617, the new compound could contribute significantly to the therapy of bone metastases by exploiting the heterodimeric dual-targeting mechanism and thereby increasing avidity as discussed above. This is particularly the case when PSMA expression in metastases is low or absent and therapy with [^177^Lu]Lu-PSMA-617 or other PSMA-based therapeutics is not possible. In addition, it enables a simpler therapeutic approach. Likewise, PSMA-negative metastases cannot be “overlooked.” The possibility to target via both target vectors with a single theranostic radiotracer could improve patient management. Although the compound in the first studies is a promising compound for theranostics of PCa related bone metastases, further investigation and optimisation is needed to improve the radiotracer.

## Data Availability Statement

The raw data supporting the conclusions of this article will be made available by the authors, without undue reservation.

## Ethics Statement

All animal experiments were approved by the Ethical Committee of the State of Rhineland Palatinate (according to §8 Abs. 1 Tierschutzgesetz, Landesuntersuchungsamt) and performed in accordance with relevant federal laws and institutional guide-lines. Approval Nr. 23 177-07/G 21-1-022.

## Author Contributions

TG, HL, and FR planned the study. TG performed organic synthesis, radiolabelling, *in vivo* stability, lipophilicity studies, HAP binding studies, and analysis of the obtained data. HL was responsible for LNCaP cell cultivation, *in vitro* binding affinity assay and its analysis, and was in charge of analysis of the obtained data of the animal study. Animal study were performed by NB, HL, and TG. TG and HL wrote the manuscript. MM and FR supervised the project. All authors reviewed the manuscript, contributed to the article, and approved the submitted version.

## Funding

The open access publication fees were funded by the Johannes Gutenberg University Mainz, Germany.

## Conflict of Interest

The authors declare that the research was conducted in the absence of any commercial or financial relationships that could be construed as a potential conflict of interest.

## Publisher's Note

All claims expressed in this article are solely those of the authors and do not necessarily represent those of their affiliated organizations, or those of the publisher, the editors and the reviewers. Any product that may be evaluated in this article, or claim that may be made by its manufacturer, is not guaranteed or endorsed by the publisher.
